# A laboratory-based cross-sectional study about helicobacter pylori infection and diabetes

**DOI:** 10.22088/cjim.15.1.20

**Published:** 2024

**Authors:** Mehrdad Haghighi, Mehdi Goudarzi, Abdolreza Babamahmoodi

**Affiliations:** 1 Infectious Diseases and Tropical Medicine Research Center, Shahid Beheshti University of Medical Sciences, Tehran, Iran; 2 Department of Microbiology, School of Medicine, Shahid Beheshti University of Medical Sciences, Tehran, Iran; 3 Antimicrobial Resistance Research Center, Communicable Diseases Institute, Mazandaran University of Medical Sciences, Sari, Iran

**Keywords:** HbA1c, Helicobacter pylori infection, Type 2 diabetes

## Abstract

**Background:**

Despite the numerous articles discussing the relationship between diabetes mellitus type 2 (DMT2) and chronic Helicobacter pylori (H. pylori) infection the results have been inconsistent, necessitating further research. This study investigated the coexistence of Helicobacter pylori infection and DMT2.

**Methods:**

We conducted a study in selected laboratories in Tehran, measuring the H.Pylori stool antigen (HpSA) in individuals referred by physicians for a glycosylated hemoglobin A1c (HbA1c) test.

**Results:**

Out of the 2500 patients who were referred to randomly selected laboratories, a total of 2025 (81%) patients had serum HbA1c levels above 6.5%. of 2025 patients with HbA1c levels above 6.5%, 1321 (52.84%) had HpSA in their stool. No significant gender difference was observed, with a mean age ± SD, 48.65 ± 7.55. HpSA was positive in 52.84% of the DM group, while in the non-DM group, HpSA was positive in 37.36% of cases. Fecal antigen titers are not related to gender (P = 0.274) but are related to age (r = 0.213, P=0.034).

**Conclusion:**

Long-term infection with Helicobacter pylori may be significantly associated with elevated HgA1c.Testing for H. pylori infection, regular monitoring of blood sugar and HbA1c levels in high-risk people can prevent DMT2.

DMT2 is a systemic disease characterized by hyperglycemia, which is caused by a defect in the metabolism of carbohydrates, proteins, and fats. This defect can be due to an absolute or partial insulin deficiency and/or a defect in its function. According to the World Health Organization (WHO), DMT2 is the most common type of DM and affects about 90% of individuals with diabetes. DMT2 is a prevalent disease that kills about 3.8 million people worldwide (1).

Since Warren and Marshall introduced H. pylori in 1983, this bacterium has proven to play a role in various diseases (2). The antibodies against this bacterium have also been linked to various diseases, such as diabetes (3). Recent studies have also shown that the immunological effects of H. pylori can lead to problems in the immune and endocrine systems (4, 5). Some systematic reviews and meta-analyses were conducted to assess the global prevalence of H. pylori infection. The findings revealed that more than half of the world's population is infected (6). H. pylori infection can cause ischemic manifestations within and outside of the human gastrointestinal tract. The potential effects of H. pylori in causing ischemic events in the cardiovascular system, skin, liver, and pancreas, as well as the nervous system and malignancies (7), have been discussed in various articles (8).

The researchers conducted a systematic review showing that the total number of H. pylori infections in diabetic patients was greater than 50%, regardless of the type of diabetes (9). WHO officially recommends an HbA1c level greater than 6.5% as the diagnostic cut-off point for diabetes (10). 

The HpSA using the monoclonal enzyme-immunoassay (EIA) method has demonstrated high accuracy in diagnosing infection (11) .Almost all of the large studies conducted on this subject suggest that further research is necessary to ascertain the definitive role of H. Pylori in DMT2. Therefore, we have designed and conducted this research. Understanding the role of H. pylori in DMT2 can be beneficial for prognosis, treatment, policy-making, and disease management.

## Methods

**Ethics Statement:** The protocol for this study was approved by the Ethics Committee of Shahid Beheshti University of Medical Sciences, Tehran, Iran. We have no conflict of interest issues. This study was done with the financial support of Shahid Beheshti University of Medical Sciences. This study was approved by the Research Ethics Committee IR.SBMU.RETECH.REC.1400.198.

To study and evaluate the relationship between DMT2 and H. pylori, we performed a descriptive cross-sectional study to evaluate HpSA in patients referred for HbA1C measurement. We selected several laboratories in Tehran to reach diabetics. We included persons over 25 years of age who were referred by a physician for the initial diagnosis of diabetes. We randomly selected referral laboratories, and sampling continued until we reached 2500 samples. The study lasted from 2020 to the first quarter of 2022.

**Inclusion criteria: **Age over 25 years, and HbA1c greater than 6.5% (48 mmol/mol).

**Exclusion criteria:**

· Patients who have been taking PPIs, bismuth, and H2 receptor antagonists for the past month

· Patients who have been taking antibiotics for the past 3 months

· Patients who have been taking antacids for the past two days prior to sampling

· History of hemoglobinopathy

· History of taking antidiabetic drugs for any reason

· Normal thyroid hormone levels

· No sickle cell or asplenia

· No history of chronic renal failure

· No anemia

**Laboratory methods:** Stool samples were obtained simultaneously from all individuals who came for diabetes tests and were sent to the laboratory for further analysis. HpSA titers were evaluated using the enzyme-linked immunosorbent assay (ELISA) technique.

Premier Platinum HpSA Plus is an enzyme immunoassay for the detection of Helicobacter pylori stool antigens. It is a highly sensitive test to detect active H. pylori infection. These products are offered by Launch Diagnostics, Meridian Bioscience, Szabo-Scandic, Medline Industries, Greyline Medical, and Fisher Scientific. The assay is an in vitro qualitative procedure for detecting H. pylori antigens in human stool samples. It can distinguish between active and non-active infections. The test is user-friendly and provides accurate results. Two certified systems of the National Glycohemoglobin Standardization Program (NGSP), D10 and COBAS INTEGRA 400 were considered acceptable methods for measuring HbA1C.

## Results

Out of the 2500 patients who were referred to randomly selected laboratories, a total of 2025 (81%) patients had serum HbA1c levels above 6.5%. Additionally, out of the 2025 people who had HbA1c levels above 6.5%, 1321 (52.84%) patients had HpSA in their stool. No significant gender difference was observed, with a mean age of 48.65 ± 7.55. HpSA was positive in 71.16% of the diabetics group, while in the non-diabetic group, HpSA was positive in 37.36% of cases. Stool antigen titers have not association with gender (P = 0.274), but they were correlated with age (r = 0.213, P= 0.034). H. Pylori infection was significantly associated with high levels of HbA1c and DMT2 in participants above 55 years old (P = 0.001). In the study group, 1,875 people (75%) were urban, and 625 people (25%) were rural. There were 1300 women (52%) and 1200 men (48%). Education level, gender, and residence did not have a significant effect on H. pylori infection. In rural areas, there is a positive correlation between increasing age (from 55 years of age) and H. pylori infection and HbA1c level. However, in the urban population, only a positive association was observed between aging and H. pylori infection, and hemoglobin level was not associated with the first two variables. 

## Discussion

Although this study suggests that people infected with H. pylori are more likely to develop diabetes than those who are not, scientists still do not have a clear consensus. There is controversy about the relationship between H. pylori infection and DMT2, as some studies have shown that the infection is more common in people with diabetes (12), while others report no difference (13). Today, there is a growing number of proponents of the theory linking H. pylori infection and DMT2. Systematic reviews, meta-analyses (14), and even big data analyses on electronic health records (15) confirm this relationship.In diagnosing and confirming H. pylori infection, several studies have demonstrated that the monoclonal analysis of fecal antigen has high sensitivity and specificity, making it superior to the serum antibody method (16). Chen. J et al. conducted a meta-analysis study and suggested that there may be an association between H. pylori infection and HbA1C levels in diabetics (17). Mansouri. K et al. In a meta-analysis conducted in Iran, the researchers highlighted the relationship between the occurrence of H. pylori and DMT2. They indicated the high prevalence of this infection among diabetics. That’s why, they recommended that treatment and eradication of this bacterium should be taken into consideration for diabetics (18).

Chen et al. conducted a study to investigate the relationship between stomach H. pylori colonization and HbA1C levels. They concluded that this association is significant (19). In another study, researchers also found that chronic H. pylori infection was significantly associated with decreased insulin secretion and higher HbA1c levels (20). In a study of more than 20,000 participants, Kato et al.’s H. pylori infection is now associated with an increased risk of diabetes based on the discovery of serum antibodies. However, this increased risk was not observed in participants after eradication (21). Chen, YY et al. in a cohort study revealed that H. pylori increases the risk of metabolic syndrome and diabetes (22).There are some papers that suggest there is no significant relationship between H. pylori infection and diabetes mellitus. Here are some examples from the search results:Man, S. et al. reported There was no significant association between H. pylori infection and DMT2 in the entire study population. However, subgroup analysis emphasized that H. pylori infection may be associated with an increased risk of DMT2 in women (23). 

One paper stated that it is not clear whether a significant relationship between H. pylori and diabetes mellitus exists, and the mechanisms underlying any potential relationship are not well understood (24). A study on 195 diabetic patients showed poor or no association between H. pylori infection and DM (1). Overall, while some researchers suggest that there may be a co-relationship between H. pylori infection and diabetes mellitus, there are also papers suggesting there is no significant relationship between the two. Based on our research and analyses of several studies, we have concluded that H. pylori infection is more common in diabetics. Furthermore, there is a significant and remarkable association between the presence of dm and the risk of h. pylori infection among the elderly, especially in those older than 55 years. There was no significant difference in the distribution of H. pylori infection in the male and female populations. Detection of H. pylori infection and monitoring of blood glucose and hba1c levels can prevent dmt2. We propose therapists take into consideration the possibility of an association between Helicobacter pylori infection and diabetes and take steps to prevent it by annually monitoring the fecal antigen level of this bacterium in patients who are at risk for developing the disease.
